# Supplementary material for: Broadband microwave detection using electron spins in a hybrid diamond-magnet sensor chip
Source: Nat Commun. 2023 Jan 30;14:490. doi: 10.1038/s41467-023-36146-3 (PMC9887009; doi:10.1038/s41467-023-36146-3)
Supplement: Supplementary file 1 — Supplementary Information [file 41467_2023_36146_MOESM1_ESM.pdf]

Supplementary Information

Broadband microwave detection using electron spins in a hybrid diamond-magnet sensor chip

Joris J. Carmiggelt, Iacopo Bertelli, Roland W. Mulder, Annick Teepe, Mehrdad Elyasi, Brecht G. Simon, Gerrit E. W. Bauer, Yaroslav M. Blanter, Toeno van der Sar

Correspondence to: T.vanderSar@tudelft.nl

Supplementary Note 1 - Derivation of the spin-wave dispersion for bias fields along the NV axis

Here we derive the spin-wave dispersion for a magnetic film in the xy-plane with perpendicular magnetic anisotropy (PMA) and a magnetic bias field $\mathbf{B}_B$ in an arbitrary direction. The dispersion is given by the poles of the transverse magnetic susceptibility[1,2] that relates the transverse magnetization to a drive field $\mathbf{B}_{\mathrm{AC}}$. We derive the magnetic susceptibility from the Landau-Lifshitz-Gilbert (LLG) equation that describes the dynamics of the unit magnetization vector $\mathbf{m}$

$$\dot{\mathbf{m}} = -\gamma \mathbf{m} \times \mathbf{B} - \alpha_{\mathrm{G}} \dot{\mathbf{m}} \times \mathbf{m}, \tag{1.1}$$

where α_{G} is the Gilbert damping and the "overdot" denotes the time derivative. We solve this equation in the (x', y', z') magnet frame that is tilted with respect to the (x, y, z) lab frame by an angle θ_0, such that the equilibrium magnetization points in the $\hat{z}'$ direction and the $\hat{y}^{(\prime)}$ axes overlap. $\mathbf{B} = \mathbf{B}_{\mathrm{eff}} + \mathbf{B}_{\mathrm{AC}}$, with $\mathbf{B}_{\mathrm{eff}}$ the effective magnetic field as derivative of the magnetic free energy density F

$$B_{\mathrm{eff},\alpha'} = -\frac{1}{M_{\mathrm{s}}} \frac{\partial F}{\partial m_{\alpha'}}, \tag{1.2}$$

where M_{s} is the saturation magnetization and $\alpha' \in \{x', y', z'\}$ indicates the vector components in the magnet frame. The free energy density includes the Zeeman energy, the demagnetizing field $\mathbf{B}_{\mathrm{d}}$, the PMA energy F_{A}, and the exchange interaction

$$F = -M_s \mathbf{m} \cdot \left(\mathbf{B}_B + \frac{\mathbf{B}_{\mathrm{d}}}{2} \right) + F_{\mathrm{A}} + \frac{D}{2} \sum_{\alpha,\beta=x,y,z} \left(\frac{\partial m_{\alpha'}}{\partial \beta} \right)^2. \tag{1.3}$$

In the magnet frame

$$F_{\mathrm{A}} = \frac{K}{2} m_z^2 = \frac{K}{2} (\sin\theta_0\, m_{x'} + \cos\theta_0\, m_{z'})^2, \tag{1.4}$$

such that the x' and z' components of the anisotropy effective field are

$$B_{\mathrm{A},x'} = -\frac{1}{M_{\mathrm{s}}} \frac{\partial F}{\partial m_{x'}} = -\frac{K}{M_s} (\sin^2\theta_0\, m_{x'} + \cos\theta_0 \sin\theta_0\, m_{z'}), \tag{1.5}$$

$$B_{\mathrm{A},z'} = -\frac{1}{M_\mathrm{s}}\frac{\partial F}{\partial m_{z'}} = -\frac{K}{M_s}(\cos\theta_0 \sin\theta_0\, m_{x'} + \cos^2\theta_0\ m_{z'}). \tag{1.6}$$

The contributions of the Zeeman-, demagnetizing- and exchange energy to $\mathbf{B}_\mathrm{eff}$ have been derived in Refs.[1,2].

In linear response with $m_{z'} \approx 1$, the LLG equation describes the transverse magnetization dynamics. In the frequency domain it reads

$$-i\omega m_{x'} = -\gamma\left(m_{y'}B_{z'} - B_{y'}\right) + i\alpha_\mathrm{G}\omega m_{y'}, \tag{1.7}$$

$$-i\omega m_{y'} = \gamma(m_{x'}B_{z'} - B_{x'}) - i\alpha_\mathrm{G}\omega m_{x'}, \tag{1.8}$$

where ω is the angular frequency. Substituting the components of the effective magnetic field and rewriting the equations in matrix form,

$$\begin{pmatrix} \omega_2 - i\alpha_\mathrm{G}\omega & -\omega_1 + i\omega \\ -\omega_1 - i\omega & \omega_3 - i\alpha_\mathrm{G}\omega \end{pmatrix}\begin{pmatrix} m_{x'} \\ m_{y'} \end{pmatrix} = \gamma\begin{pmatrix} B_{\mathrm{AC},x'} \\ B_{\mathrm{AC},y'} \end{pmatrix}, \tag{1.9}$$

where

$$\omega_0 = -(\omega_M - \omega_K)\cos^2\theta_0 + \omega_B\cos(\theta_B - \theta_0) + \omega_D k^2, \tag{1.10}$$

$$\omega_1 = \omega_M f \sin\phi\cos\phi\cos\theta_0, \tag{1.11}$$

$$\omega_2 = \omega_0 + \omega_M f(\cos^2\phi\cos^2\theta_0 - \sin^2\theta_0) + (\omega_M - \omega_K)\sin^2\theta_0, \tag{1.12}$$

$$\omega_3 = \omega_0 + \omega_M f \sin^2\phi, \tag{1.13}$$

and $\omega_B = \gamma B_B$, $\omega_M = \gamma\mu_0 M_\mathrm{s}$, $\omega_D = \frac{\gamma D}{M_\mathrm{s}}$ and $\omega_K = \frac{\gamma K}{M_\mathrm{s}}$. μ_0 is the vacuum permeability, $k = |\mathbf{k}|$ is the modulus of the wavevector along an angle ϕ with respect to the in-plane projection of the magnetization, θ_B is the angle of the magnetic bias field with respect to the plane normal (z axis), and $f = 1 - \frac{(1-e^{-kt})}{kt}$ depends on the film thickness t. By inverting the matrix in Eq. (S1.9), we obtain the transverse magnetic susceptibility, which is singular when

$$(\omega_2 - i\alpha_\mathrm{G}\omega)(\omega_3 - i\alpha_\mathrm{G}\omega) - \omega_1^2 - \omega^2 = 0. \tag{1.14}$$

Assuming $\alpha_\mathrm{G} \ll 1$, the real part of the solutions of this quadratic equation gives the spin-wave dispersion as a function of k

$$\omega^2 = \omega_2\omega_3 - \omega_1^2. \tag{1.15}$$

The theoretical lines in Figs. 2 and 4 in the main text are based on Supplementary Equation 1.15. We assume that the field is applied parallel to the NV axis, such that $\theta_B = 54.7°$, with in-plane projection along the stripline. We consider only spin waves with $\phi = \pi/2$, since these are most efficiently excited by our 150-micron-long stripline. θ_0 minimizes the free energy density and is found by numerically solving $\frac{\partial F}{\partial \theta_0} = 0$. The ferromagnetic resonance (FMR) frequency corresponds to $k = 0$. Supplementary Table 1 states the values of the saturation magnetization, exchange and uniaxial anisotropy constants for different magnetic materials used for calculating the spin-wave dispersions in Supplementary Fig. 7.

Supplementary Note 2 - Dependence of the detection bandwidth on the microwave drive field

For efficient frequency conversion, the microwaves should excite propagating spin waves with a significant amplitude. The spin-wave excitation efficiency depends on the microwave power and the spatial mode overlap between the drive field and the spin waves[3]. In our experiment, a 5-micron-wide stripline creates an inhomogeneous microwave drive field with a sinc-like amplitude in k-space (Supplementary Fig. 1a). The efficiency drops with decreasing wavelength with nodes at $\lambda = w/n$, where w is the stripline width and n is an integer.

To characterize the dependence on the drive field, we measure the bandwidth induced by four-wave mixing as a function of the pump power (Supplementary Fig. 1b). As expected, the bandwidth increases with microwave power. The photoluminescence contrast is suppressed at spin-wave frequencies that correspond to the nodes of the drive field in Supplementary Fig. 1a (colored dashed lines). The frequencies of these modes agree with the spin-wave dispersion derived in the previous section (Supplementary Equation 1.15). The spin-wave excitation

antenna is therefore an important design parameter for hybrid diamond-magnet microwave sensors.

Supplementary Note 3 - Comparison between the idler-driven Rabi frequency and dynamical Stark shift

A strong microwave field detuned by δf from the NV ESR frequency (f_{NV}), causes the latter to shift, an effect known as the AC (or dynamical) Stark shift[4]. The Stark shift increases with drive power and is inversely proportional to δf, which allows detecting the presence of an off-resonant microwave signal. We show here that the idler-driven Rabi frequency resulting from four-spin-wave mixing is about an order magnitude larger than the Stark shift at the same off-resonant drive power.

We measure the Stark shift via pump-probe microwave spectroscopy. The high-power pump is detuned from f_{NV} by 10-1000 MHz, while a low-power probe measures the ESR frequency. We determine the Stark shift for every detuning by measuring the ESR frequency with and without pump (blue data in Supplementary Fig. 4a).

Next, we measure Rabi oscillations using the four-spin-wave mixing technique. We extract the Rabi frequency for signal spin waves detuned from 10 to 710 MHz (red data in Supplementary Fig. 4a). We attribute the small oscillations in the Rabi frequency and Stark shift to frequency-dependent (cable) resonances in the microwave transmission of the stripline. Supplementary Fig. 4b shows that the Rabi frequencies are larger than the Stark shift by about an order of magnitude over the measurement range.

Supplementary Note 4 - Eight-modes model

Here we describe the details of the model for the spin-wave dynamics under a two-tone drive used to calculate the idler amplitude as a function of pump and signal power, as plotted in Fig. 3E in the main text.

Supplementary Fig. 5 shows the spin-wave dispersion of a YIG film for $\theta_{\mathbf{k}} = 0$ (blue line) and $\theta_{\mathbf{k}} = \pi/2$ (black line), where $\theta_{\mathbf{k}}$ is the angle between the in-plane spin-wave wavevector $\mathbf{k}$ and the static magnetization for the parameters in Supplementary Table 1. Since the out-of-plane component of the applied bias field $\mathbf{B}_{\mathrm{NV}}$ is small compared to the demagnetizing field of ~ 178 mT in YIG, we assume that the static magnetization lies in-plane along $\hat{z}$ ($\hat{x}$ is the out-of-plane axis), parallel to $B_{\hat{z}}$, the in-plane component of $\mathbf{B}_{\mathrm{NV}}$. The long stripline along $\hat{z}$ excites signal and pump spin waves with $\theta_{\mathbf{k}} = \pi/2$. Conservation of momentum dictates that the two created idler spin waves also lie on the $\theta_{\mathbf{k}} = \pi/2$ branch with wavevectors $\mathbf{k}_{\mathrm{i}} = 2\mathbf{k}_{\mathrm{p}} - \mathbf{k}_{\mathrm{s}}$ and $\mathbf{k}_{\mathrm{i}'} = 2\mathbf{k}_{\mathrm{s}} - \mathbf{k}_{\mathrm{p}}$ (Supplementary Fig. 5).

When the pump mode is strongly driven beyond a certain threshold, the four magnon scattering term in the spin-wave Hamiltonian $c^{\dagger}_{\mathbf{k}_{\mathrm{p}}} c^{\dagger}_{\mathbf{k}_{\mathrm{p}}} c_{\mathbf{k}'} c_{\mathbf{k}''}$ leads to a Suhl instability. Here $c^{(\dagger)}_{\mathbf{k}}$ is the annihilation (creation) operator for a magnon with wavevector $\mathbf{k}$,which is normalized by $\sqrt{S}$, where $S = V s_n / V_n$ is the total number of spins, V is the volume, s_n is the number of spins per unit cell, and V_n is the unit cell volume. A specific pair of magnons wins the 'instability competition', $\mathbf{k}' = \mathbf{k}_{\mathrm{p},1}$ and $\mathbf{k}'' = \mathbf{k}_{\mathrm{p},2} = 2\mathbf{k}_{\mathrm{p}} - \mathbf{k}_{\mathrm{p},1}$, which we call the 'efficient Suhl pair' of the pump mode. The efficient Suhl pair for the signal $\mathbf{k}_{\mathrm{s},1}$ and $\mathbf{k}_{\mathrm{s},2}$ should also be considered when its mode amplitude is sufficiently large. We disregard cascades that lead to the weak higher-order idlers in Fig. 2f of the main text, as well as the Suhl pairs of the idlers that are safely below their instability threshold at the presently applied powers. A minimal model should therefore include the eight modes indicated in Supplementary Fig. 5.

The efficient pump and signal pairs can be identified from the threshold amplitude of the pump (signal) mode $x = |\alpha_{\mathrm{p(s)}}|^2$ above which the Suhl instability leads to $\{\mathbf{k}', \mathbf{k}''\}$ pairs, which solve[5]

$$\left(\mathcal{D}^{\mathrm{Suhl}}_{\mathrm{p(s)};\mathbf{k}';\mathbf{k}''}{}^2 - \mathcal{D}^{\mathrm{CK}}_{\mathrm{p(s)};\mathbf{k}'}{}^2\right) x^2 - 2\Delta\mathcal{D}^{\mathrm{CK}}_{\mathrm{p(s)};\mathbf{k}'} x - \xi^2 - \Delta^2 = 0, \tag{4.1}$$

where $\Delta = \omega_{\mathrm{p(s)}} - (\omega_{\mathbf{k}'} + \omega_{\mathbf{k}''})/2$, with ω an angular frequency, and ξ is a dissipation rate chosen here to be 10 MHz for all modes. $\mathcal{D}^{\mathrm{Suhl}}_{\mathrm{p(s)};\mathbf{k}';\mathbf{k}''}$ ($\mathcal{D}^{\mathrm{CK}}_{\mathrm{p(s)};\mathbf{k}'}$) is the matrix element for the scattering process $c^\dagger_{\mathbf{k}_{\mathrm{p(s)}}} c^\dagger_{\mathbf{k}_{\mathrm{p(s)}}} c_{\mathbf{k}'} c_{\mathbf{k}''}$ ($c^\dagger_{\mathbf{k}_{\mathrm{p(s)}}} c_{\mathbf{k}_{\mathrm{p(s)}}} c^\dagger_{\mathbf{k}'} c_{\mathbf{k}'}$) in the Hamiltonian[6]. First, we numerically calculate the threshold amplitude $|\alpha_{\mathrm{p(s)}}|^2$ as a function of $|\mathbf{k}'|$ and $\theta_{\mathbf{k}'}$ as in Supplementary Fig. 6a. We identify the minimum threshold amplitude in the ($|\mathbf{k}'|$,$\theta_{\mathbf{k}'}$) plane of Supplementary Fig. 6a as a function of modulus $|\mathbf{k}'|$ in Supplementary Fig. 6b. The corresponding $\theta_{\mathbf{k}'}$ and spin-wave pair frequencies are shown in Supplementary Figs. 6b and 6c, respectively. The spin-wave pair with the lowest threshold amplitude – the effective pump (signal) Suhl pair – turns out to be at angles $\theta_{\mathbf{k}'}$ far from $\pi/2$ (as indicated by the vertical dashed lines in Supplementary Fig. 6b-c).

Our model Hamiltonian reads

$$\begin{aligned} H = \sum_X \omega_X c^\dagger_X c_X + \Big[&\mathcal{D}^{\mathrm{Suhl}}_{\mathrm{p;p,1;p,2}} c^\dagger_{\mathbf{k}_\mathrm{p}} c^\dagger_{\mathbf{k}_\mathrm{p}} c_{\mathbf{k}_{\mathrm{p},1}} c_{\mathbf{k}_{\mathrm{p},2}} + \\ &\mathcal{D}^{\mathrm{Suhl}}_{\mathrm{p;s;i}} c^\dagger_{\mathbf{k}_\mathrm{p}} c^\dagger_{\mathbf{k}_\mathrm{p}} c_{\mathbf{k}_\mathrm{s}} c_{\mathbf{k}_\mathrm{i}} + \mathcal{D}^{\mathrm{Suhl}}_{\mathrm{s;s,1;s,2}} c^\dagger_{\mathbf{k}_\mathrm{s}} c^\dagger_{\mathbf{k}_\mathrm{s}} c_{\mathbf{k}_{\mathrm{s},1}} c_{\mathbf{k}_{\mathrm{s},2}} + \\ &\mathcal{D}^{\mathrm{Suhl}}_{\mathrm{s;p;i'}} c^\dagger_{\mathbf{k}_\mathrm{s}} c^\dagger_{\mathbf{k}_\mathrm{s}} c_{\mathbf{k}_\mathrm{p}} c_{\mathbf{k}_{\mathrm{i}'}} + \mathrm{H.c.}\Big] + \\ &\sum_X \left[\mathcal{D}^{\mathrm{SK}}_X c^\dagger_X c_X c^\dagger_X c_X + \sum_Y \mathcal{D}^{\mathrm{CK}}_{X;Y} c^\dagger_X c_X c^\dagger_Y c_Y\right] + \\ &E'_\mathrm{p}\left(e^{-i\omega_\mathrm{p} t} c^\dagger_\mathrm{p} + \mathrm{H.c.}\right) + E'_\mathrm{s}\left(e^{-i\omega_\mathrm{s} t} c^\dagger_\mathrm{s} + \mathrm{H.c.}\right). \end{aligned} \tag{4.2}$$

Here $X, Y \in \{\mathrm{p; s; i; i'; p,1(2); s,1(2)}\}$, and H.c. denotes the Hermitian conjugate. E'_s and E'_p are the drive amplitudes of the signal and pump modes, respectively, which are related to the excitation power of the microstrip by (cf. Supplementary Fig. 1a),

$$P_{\mathrm{p(s)}} = E_{\mathrm{p(s)}}^2 Z(\omega_{\mathrm{p(s)}}) \left[\frac{\mu_0 \gamma \left(e^{-|\mathbf{k}_{\mathrm{p(s)}}|d} - 1 \right) \sin\left(\frac{|\mathbf{k}_{\mathrm{p(s)}}| W}{2} \right)}{W d |\mathbf{k}_{\mathrm{p(s)}}|^2} \right]^{-2} . \quad (4.3)$$

Here, μ_0 is the vacuum permeability, d is the thickness of the stripline and W is its width, $Z(\omega)$ is the impedance at ω, and we assumed $\theta_{\mathbf{k}_{\mathrm{p(s)}}} = \pi/2$. We adopt $Z(\omega_{\mathrm{p(s)}}) = 50\ \Omega$, $d = 200$ nm, $W = 5$ µm, $\mathrm{E}_{\mathrm{p(s)}} = \mathrm{E}'_{\mathrm{p(s)}}\sqrt{L}$, where $L \sim W$ is the length of the excited part of the sample, $\omega_{\mathrm{p}}/2\pi = 2.2$ GHz, $\omega_{\mathrm{s}}/2\pi = 2.32$ GHz and $B_{\hat{z}} = 23$ mT, corresponding to $B_{\mathrm{NV}} \sim 28$ mT. From the four-magnon-scattering parameters $\mathcal{D}^{\mathrm{Suhl}}_{\mathrm{p;p,1;p,2}}/\mathcal{D}^{\mathrm{Suhl}}_{\mathrm{p;s;i}} \sim \mathcal{D}^{\mathrm{Suhl}}_{\mathrm{s;s,1;s,2}}/\mathcal{D}^{\mathrm{Suhl}}_{\mathrm{s;p;i'}} \sim 10$, and $\mathcal{D}^{\mathrm{Suhl}}_{\mathrm{s;p;i'}} \sim \mathcal{D}^{\mathrm{Suhl}}_{\mathrm{p;s;i}} = -7.2$ GHz, and $\mathcal{D}^{\mathrm{SK}}_{\mathrm{p(s,i,i')}} \sim \mathcal{D}^{\mathrm{CK}}_{\mathrm{p(s,i,i'),p(s,i,i')}} \sim \mathcal{D}^{\mathrm{Suhl}}_{\mathrm{p;s;i}}$, and $\mathcal{D}^{\mathrm{SK}}_{\mathrm{s,1(s,2;p,1;p,2)}} \sim \mathcal{D}^{\mathrm{CK}}_{\mathrm{p(s),s,1(s,2;p,1;p,2)}} \sim \mathcal{D}^{\mathrm{Suhl}}_{\mathrm{p;p,1;p,2}}$ we calculate the mean field amplitude of the idler mode $\langle c^\dagger_{\mathbf{k}_\mathrm{i}} c_{\mathbf{k}_\mathrm{i}} \rangle = |\alpha_\mathrm{i}|^2$ as a function of P_s and P_p. In Fig. 3e of the main text we plot $|\alpha_\mathrm{i}|$, since it is linearly proportional to the idler-driven Rabi frequency of the NV center[3].

We find an idler amplitude (and thus a Rabi frequency) that initially grows as a function of pump and signal power. However, above the Suhl instability thresholds, the amplitude of the idler mode decreases due to the newly opened dissipation channels, as observed in the experiments in Fig. 3d of the main text. Since $\langle \mathrm{c}^\dagger_X \mathrm{c}_X \rangle \propto E^2_{\mathrm{s(p)}}/\xi^2$, ξ can be scaled by q to achieve the same phase diagram for $P_{\mathrm{p(s)}}$ shifted by $20 \times \log(q)$ dBm. Our current assumption of $\xi = 10$ MHz for $\omega_X/2\pi$~2 GHz corresponds to a Gilbert damping of $\alpha_\mathrm{G} = 5 \times 10^{-3}$.

Supplementary Note 5 - Difference-frequency generation by the longitudinal component of the magnetization

In this section we demonstrate that simultaneous transverse magnetization dynamics at the signal and pump frequencies (f_s and f_p, respectively) causes a beating in the longitudinal

component at the difference frequency $|f_\mathrm{s} - f_\mathrm{p}|$. The normalized transverse magnetization m_T of two propagating circularly-polarized spin waves is the superposition

$$m_\mathrm{T} = m_\mathrm{s} e^{i(k_\mathrm{s} x - \omega_\mathrm{s} t)} + m_\mathrm{p} e^{i(k_\mathrm{p} x - \omega_\mathrm{p} t)}. \quad (5.1)$$

$k_i = 2\pi/\lambda_i$ is the wavevector of the ith spin wave, with $i \in \{\mathrm{s,p}\}$, in terms of the wavelength λ_i, $\omega_i = 2\pi f_i$ is the angular frequency and $m_i = M_i/M_\mathrm{s}$ is the normalized magnetization amplitude. The transverse x and y components are the real and imaginary parts of m_T while the normalized longitudinal component of the magnetization reads

$$m_\mathrm{L} = \sqrt{1 - |m_\mathrm{T}|^2}. \quad (5.2)$$

When driving two spin waves at frequencies ω_s and ω_p, and amplitudes m_s and m_p, the squared modulus

$$|m_\mathrm{T}|^2 = m_\mathrm{T} m_\mathrm{T}^* = m_\mathrm{s}^2 + m_\mathrm{p}^2 + 2 m_\mathrm{s} m_\mathrm{p} \cos\left(\left(k_\mathrm{s} - k_\mathrm{p}\right)x - (\omega_\mathrm{s} - \omega_\mathrm{p})t\right) \quad (5.3)$$

depends on time. For $m_i \ll 1$ the longitudinal component oscillates at the difference frequency

$$m_\mathrm{L} \propto m_\mathrm{s} m_\mathrm{p} \cos\left(\left(k_\mathrm{s} - k_\mathrm{p}\right)x - (\omega_\mathrm{s} - \omega_\mathrm{p})t\right), \quad (5.4)$$

as detected in our experiments.

Supplementary Note 6 – Extending the frequency detection range using other magnetic materials

We envisions that the microwave frequency range detectable using spin-wave mixing could be extended to the 10-100 GHz scale using materials with a larger magnetization than YIG that increases the spin-wave group velocity or crystal anisotropies that increase the spin-wave gap (Supplementary Fig. 7). The four-spin-wave mixing process, spin-wave frequency combs and oscillations of the longitudinal magnetization used in our protocols have already been observed in other magnets, such as permalloy (Py), Fe, and CoFe[7–10]. The increased spin-

wave damping in these materials compared to YIG reduces the spin-wave amplitudes, but this is partially compensated by a larger saturation magnetization that increases the stray fields.

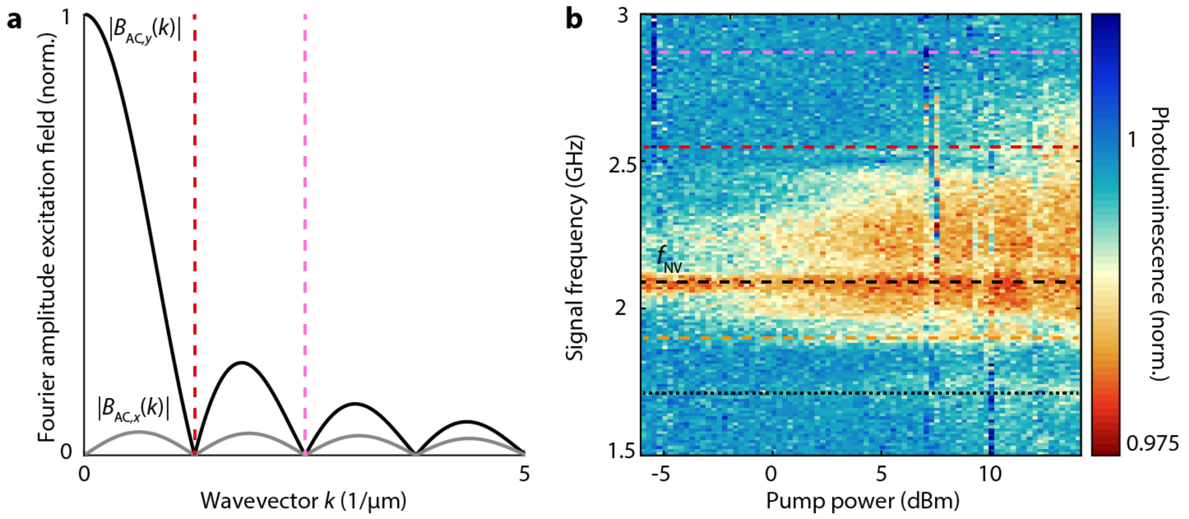

Supplementary Figure 1. The detection bandwidth is determined by the spin-wave excitation efficiency. (**a**) Normalized Fourier amplitude of the out-of-plane (x, gray) and in-plane (y, black) components of the microwave drive field $\mathbf{B}_{\mathrm{AC}}$ generated by a 5-micron-wide stripline. The colored dashed lines indicate the first two nodes in the spectrum. (**b**) Normalized NV photoluminescence induced by four-wave mixing as a function of signal frequency and pump power at a static magnetic field of $B_{\mathrm{NV}} = 28\,\mathrm{mT}$. The ESR frequency is at $f_{\mathrm{NV}} = 2.08$ GHz (black dashed line, labeled f_{NV}) and the dashed (dotted) orange (black) lines indicate the frequencies at which the signal (pump) spin waves are driving the FMR. The red and pink horizontal dashed lines indicate the frequencies of the spin waves that nominally cannot be excited by the stripline, where colors match the nodes in (a).

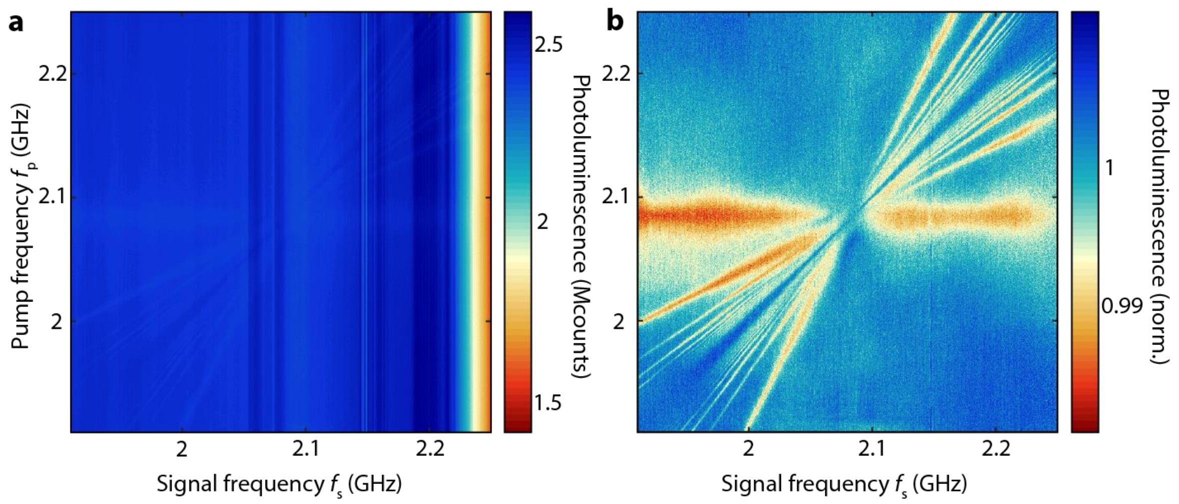

Supplementary Figure 2. Normalization procedure applied for Figs. 2f and 4d of the main text. (**a**) Raw photoluminescence data of the measurement. We attribute the fluctuations between columns to drifts of the objective focus and laser power over the course of the measurement. (**b**) By dividing the data by the median of each column the spin-wave comb is revealed. To remove the horizontal line of photoluminescence contrast caused by resonant driving at the ESR frequency $f_{\mathrm{NV}} = 2.086$ GHz, we divide the data a second time by the median of each row, resulting in Fig. 2f in the main text. The same normalization procedure was applied for Fig. 4d in the main text.

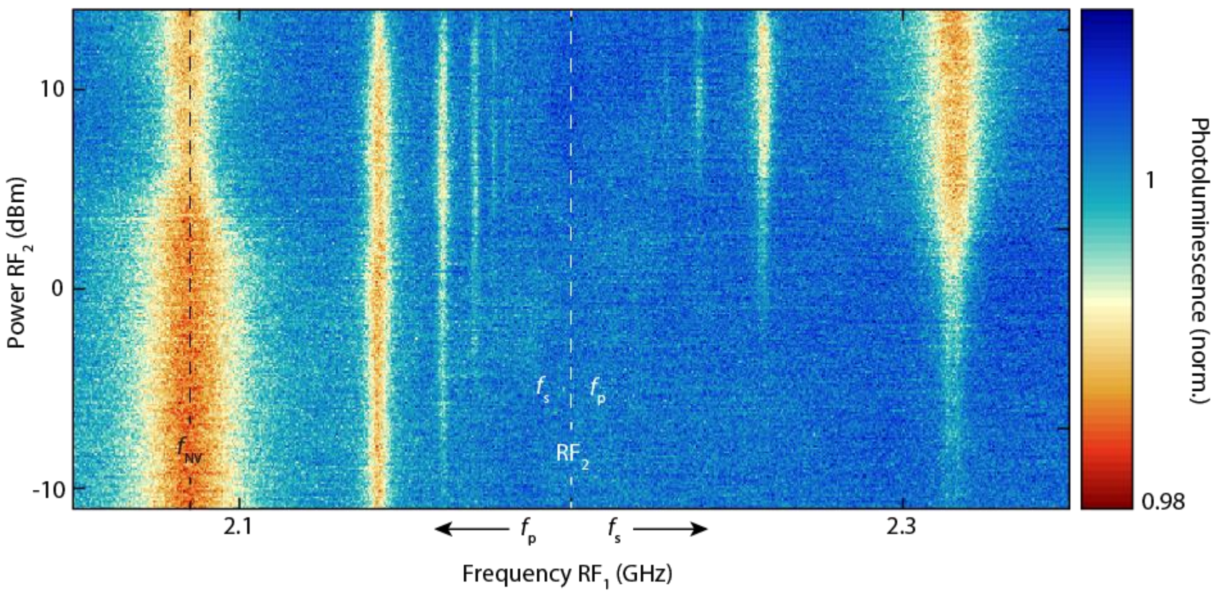

Supplementary Figure 3. Emergence of a spin-wave comb generated by two microwave drives RF$_1$ and RF$_2$. Normalized NV photoluminescence at $B_{\mathrm{NV}} = 28$ mT as a function of RF$_1$ frequency (RF$_2$ is kept at 2.2 GHz, red dashed line), and RF$_2$ power (RF$_1$ is kept at 4 dBm). An increasing number of higher-order idlers appear at increased drive power. RF$_1$ and RF$_2$ function either as pump or signal field depending on which frequency is closer to the ESR frequency $f_{\mathrm{NV}} = 2.086$ GHz, as is indicated by the labels f_{s} and f_{p} with matching colors.

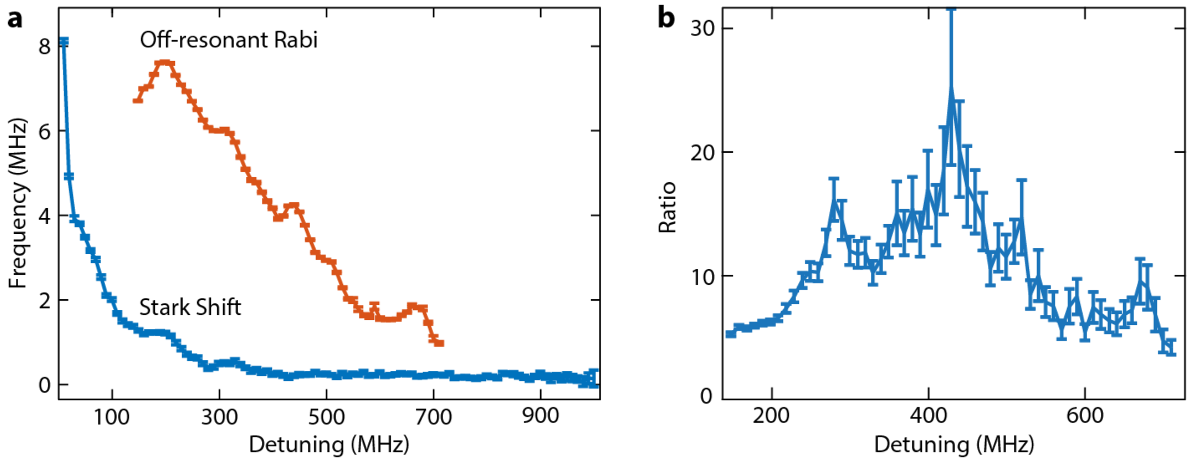

Supplementary Figure 4. Comparison of the four-spin-wave mixing and Stark shift off-resonant detection techniques. (**a**) Blue: Measured shift in NV ESR frequency due to the AC Stark effect as a function of frequency detuning of the applied drive field. Red: Frequency of the Rabi oscillations driven by the first-order idler mode using the four-spin-wave down conversion technique as a function of drive-field detuning. (**b**) Ratio between the Rabi frequency and Stark shift as a function of detuning. The measurements were carried out at a magnetic bias field of $B_{\mathrm{NV}} = 28$ mT. Error bars denote the 95% confidence interval.

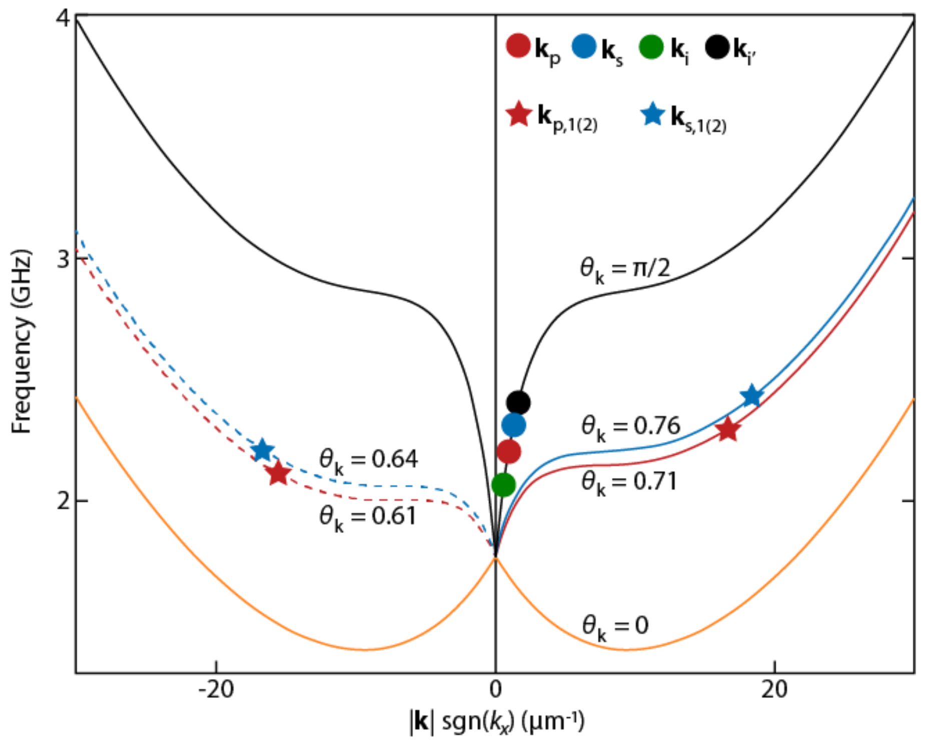

Supplementary Figure 5. Eight-modes model for the calculation of the idler amplitude. In our model we consider the signal (blue dot), pump (red dot), idlers (green and black dots), 'efficient signal Suhl instability pair' (blue stars) and 'efficient pump Suhl instability pair' (red stars) spin waves. The lines are branches of the spin-wave dispersion corresponding to different angles θ_{k} of the wavevector with respect to the static magnetization (see legend). The dispersion is symmetric upon rotations of θ_{k} by π. Calculations like those presented in Supplementary Fig. 6 lead to the wavevectors of the efficient pump and signal pairs. Here $B_{\hat{z}} = 23$ mT.

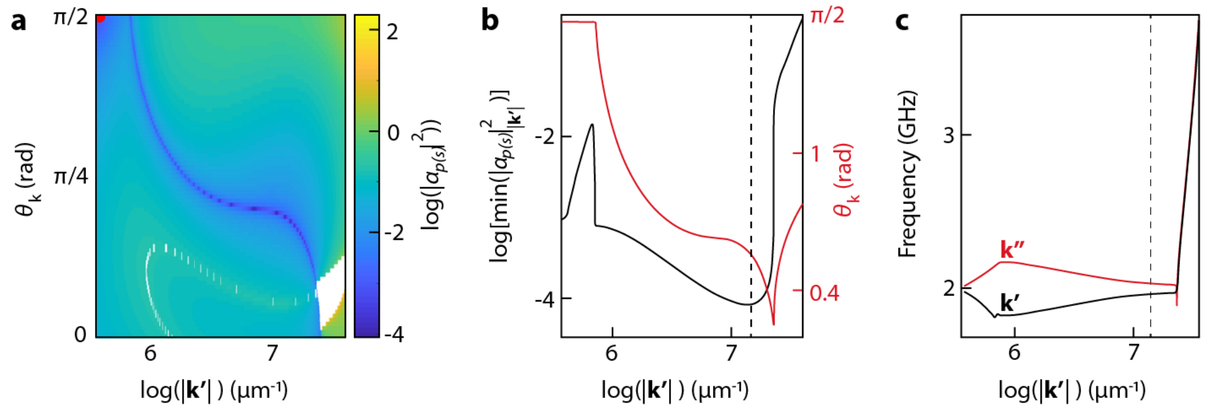

Supplementary Figure 6. Finding the efficient Suhl pair with the lowest excitation threshold. (**a**) Calculated threshold amplitude $|\alpha_{\mathrm{p(s)}}|^2$ that triggers the Suhl instability of a spin-wave pair with wavevectors $\mathbf{k}'$ and $\mathbf{k}'' = 2\mathbf{k}_{\mathrm{p(s)}} - \mathbf{k}'$ as a function of $|\mathbf{k}'|$ and $\theta_{\mathbf{k}'}$. The amplitude is normalized by the total spin S. In the white regions no momentum-conserving scattering processes can take place. Here we adopted $B_{\hat{z}} = 24$ mT, $\omega_{\mathrm{p(s)}}/2\pi = 2$ GHz and $\theta_{\mathbf{k}_{\mathrm{p(s)}}} = \pi/2$, corresponding to the red dot. (**b**) Minimal threshold amplitude as a function of $|\mathbf{k}'|$ (black line, left axis) and corresponding $\theta_{\mathbf{k}'}$ (red line, right axis). (**c**) Frequencies of the modes corresponding to the pairs in (b). The pair with the lowest threshold is indicated by the vertical dashed line in (b) and (c), and defines the 'efficient pump (signal) pair' of the Suhl instability.

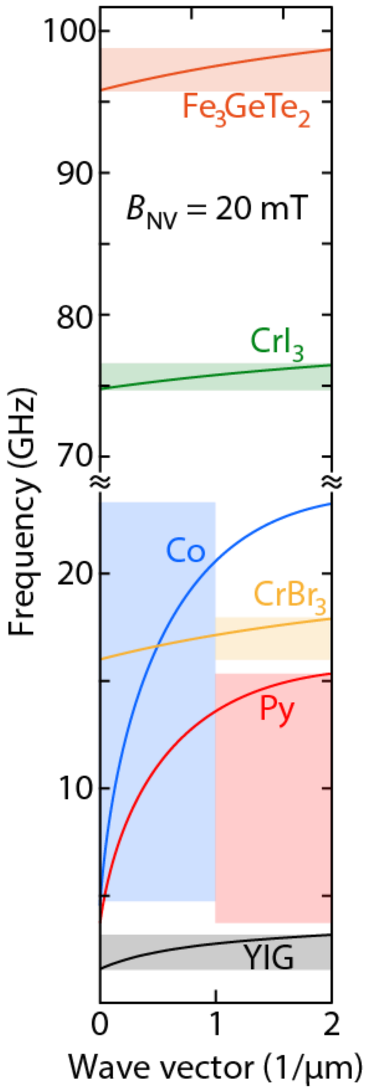

Supplementary Figure 7. Calculated spin-wave dispersion of various 100 nm-thick magnets. $B_{\mathrm{NV}} = 20$ mT. Matched color shading highlights the accessible detection bandwidth using a 500-nm-wide stripline.

Material	M_s (A/m)	D (J/m)	K (J/m^3)	Reference
YIG	$1.42 \cdot 10^5$	$4.15 \cdot 10^{-12}$	0	Ref.[3]
Permalloy (Py)	$8.46 \cdot 10^5$	$2.4 \cdot 10^{-12}$	0	
Cobalt (Co)	$13 \cdot 10^5$	$2.4 \cdot 10^{-12}$	0	
$CrBr_3$	$2.55 \cdot 10^5$	$1.2 \cdot 10^{-12}$	$2.24 \cdot 10^5$	Ref.[11]
CrI_3	$2.15 \cdot 10^5$	$1.35 \cdot 10^{-12}$	$6.30 \cdot 10^5$	Ref.[11]
Fe_3GeTe_2	$3.76 \cdot 10^5$	$9.5 \cdot 10^{-13}$	$1.46 \cdot 10^6$	Ref.[12]

Supplementary Table 1. Values of the saturation magnetization (M_s), exchange constant (*D*) and uniaxial anisotropy constant (*K*) used to calculate the spin-wave dispersions in Supplementary Fig. 7.

Supplementary References

1. Bertelli, I. *et al.* Imaging Spin-Wave Damping Underneath Metals Using Electron Spins in Diamond. *Adv. Quantum Technol.* **4**, 2100094 (2021).
2. Rustagi, A., Bertelli, I., van der Sar, T. & Upadhyaya, P. Sensing chiral magnetic noise via quantum impurity relaxometry. *Phys. Rev. B* **102**, 220403 (2020).
3. Bertelli, I. *et al.* Magnetic resonance imaging of spin-wave transport and interference in a magnetic insulator. *Sci. Adv.* **6**, eabd3556 (2020).
4. Wei, C., Windsor, A. S. M. & Manson, N. B. A strongly driven two-level atom revisited: Bloch - Siegert shift versus dynamic Stark splitting. *J. Phys. B At. Mol. Opt. Phys.* **30**, 4877–4888 (1997).
5. Elyasi, M., Saitoh, E. & Bauer, G. E. W. Stochasticity of the magnon parametron. *Phys. Rev. B* **105**, 054403 (2022).
6. Krivosik, P. & Patton, C. E. Hamiltonian formulation of nonlinear spin-wave dynamics: Theory and applications. *Phys. Rev. B* **82**, 184428 (2010).
7. Khivintsev, Y. *et al.* Nonlinear amplification and mixing of spin waves in a microstrip geometry with metallic ferromagnets. *Appl. Phys. Lett.* **98**, 042505 (2011).
8. Schultheiss, H., Vogt, K. & Hillebrands, B. Direct observation of nonlinear four-magnon scattering in spin-wave microconduits. *Phys. Rev. B* **86**, 054414 (2012).
9. Hula, T. *et al.* Nonlinear losses in magnon transport due to four-magnon scattering. *Appl. Phys. Lett.* **117**, 042404 (2020).
10. Gerrits, T., Krivosik, P., Schneider, M. L., Patton, C. E. & Silva, T. J. Direct Detection of Nonlinear Ferromagnetic Resonance in Thin Films by the Magneto-Optical Kerr Effect. *Phys. Rev. Lett.* **98**, 207602 (2007).
11. Shen, X. *et al.* Multi-domain ferromagnetic resonance in magnetic van der Waals crystals CrI_3 and $CrBr_3$. *J. Magn. Magn. Mater.* **528**, 167772 (2021).

12. León-Brito, N., Bauer, E. D., Ronning, F., Thompson, J. D. & Movshovich, R. Magnetic microstructure and magnetic properties of uniaxial itinerant ferromagnet Fe_3GeTe_2. *J. Appl. Phys.* **120**, 083903 (2016).
